# Performance of Multimodal Artificial Intelligence Chatbots Evaluated on Clinical Oncology Cases

**DOI:** 10.1001/jamanetworkopen.2024.37711

**Published:** 2024-10-23

**Authors:** David Chen, Ryan S. Huang, Jane Jomy, Philip Wong, Michael Yan, Jennifer Croke, Daniel Tong, Andrew Hope, Lawson Eng, Srinivas Raman

**Affiliations:** 1 Radiation Medicine Program, Princess Margaret Hospital Cancer Centre, Toronto, Ontario, Canada; 2 Temerty Faculty of Medicine, University of Toronto, Toronto, Ontario, Canada; 3 Department of Radiation Oncology, University of Toronto, Toronto, Ontario, Canada; 4 Division of Medical Oncology and Hematology, Department of Medicine, Princess Margaret Cancer Centre/University Health Network Toronto, Toronto, Ontario, Canada; 5 Division of Medical Oncology, Department of Medicine, University of Toronto, Toronto, Ontario, Canada

## Abstract

**Importance:**

Multimodal artificial intelligence (AI) chatbots can process complex medical image and text-based information that may improve their accuracy as a clinical diagnostic and management tool compared with unimodal, text-only AI chatbots. However, the difference in medical accuracy of multimodal and text-only chatbots in addressing questions about clinical oncology cases remains to be tested.

**Objective:**

To evaluate the utility of prompt engineering (zero-shot chain-of-thought) and compare the competency of multimodal and unimodal AI chatbots to generate medically accurate responses to questions about clinical oncology cases.

**Design, Setting, and Participants:**

This cross-sectional study benchmarked the medical accuracy of multiple-choice and free-text responses generated by AI chatbots in response to 79 questions about clinical oncology cases with images.

**Exposures:**

A unique set of 79 clinical oncology cases from JAMA Network Learning accessed on April 2, 2024, was posed to 10 AI chatbots.

**Main Outcomes and Measures:**

The primary outcome was medical accuracy evaluated by the number of correct responses by each AI chatbot. Multiple-choice responses were marked as correct based on the ground-truth, correct answer. Free-text responses were rated by a team of oncology specialists in duplicate and marked as correct based on consensus or resolved by a review of a third oncology specialist.

**Results:**

This study evaluated 10 chatbots, including 3 multimodal and 7 unimodal chatbots. On the multiple-choice evaluation, the top-performing chatbot was chatbot 10 (57 of 79 [72.15%]), followed by the multimodal chatbot 2 (56 of 79 [70.89%]) and chatbot 5 (54 of 79 [68.35%]). On the free-text evaluation, the top-performing chatbots were chatbot 5, chatbot 7, and the multimodal chatbot 2 (30 of 79 [37.97%]), followed by chatbot 10 (29 of 79 [36.71%]) and chatbot 8 and the multimodal chatbot 3 (25 of 79 [31.65%]). The accuracy of multimodal chatbots decreased when tested on cases with multiple images compared with questions with single images. Nine out of 10 chatbots, including all 3 multimodal chatbots, demonstrated decreased accuracy of their free-text responses compared with multiple-choice responses to questions about cancer cases.

**Conclusions and Relevance:**

In this cross-sectional study of chatbot accuracy tested on clinical oncology cases, multimodal chatbots were not consistently more accurate than unimodal chatbots. These results suggest that further research is required to optimize multimodal chatbots to make more use of information from images to improve oncology-specific medical accuracy and reliability.

## Key Points

**Question:**

What is the medical accuracy of multimodal artificial intelligence chatbots compared with text-only chatbots when evaluated on questions about oncology cases?

**Findings:**

In this cross-sectional study of 79 clinical oncology cases, multimodal chatbots were not consistently more accurate than unimodal chatbots in both the multiple-choice and physician-rated free-text evaluation.

**Meaning:**

These results suggest that multimodal chatbots may need additional optimization to make use of information from images in order to generate accurate responses to oncology cases.

## Teaser

This cross-sectional study assesses the ability of unimodal and multimodal artificial intelligence (AI) chatbots to generate medically accurate responses to questions about clinical oncology cases.

## Introduction

Multimodal artificial intelligence (AI) chatbots have been proposed as useful tools to answer patient questions about general medicine.^1^ Multimodal AI chatbots, compared with unimodal, text-only AI chatbots, can process complex medical image and text-based information that may further improve their accuracy as a diagnostic and management tool.^2,3^ Multimodal chatbots have demonstrated an accuracy of more than 70% when answering questions about general medicine clinical cases based on free-text and image-based input.^4^ Coupled with the adoption of electronic health record systems that can access large amounts of digital patient data, computer-assisted clinical decision support systems enabled by multimodal AI can facilitate and potentially optimize clinician decision-making with clinical knowledge and patient data.^5^ Pilot studies of AI chatbots have shown that the implementation of generative AI tools in clinical settings may relieve clinician burden and burnout.^6,7^ By integrating multimodal AI with clinical decision support systems, clinicians may interact with a chatbot interface by asking open-ended questions about the patient presentation and leverage multimodal data, including imaging, unstructured clinical text in the electronic health record, and laboratory results, to iteratively generate a recommendation for clinician interpretation.

Given the encouraging accuracy of unimodal chatbots to answer both patient^1^ and physician-generated medical questions,^8^ there remains the need to compare the medical accuracy of unimodal and multimodal AI chatbots to evaluate the clinical utility of image-processing modalities. However, the difference in medical accuracy of multimodal and text-only chatbots in addressing questions about clinical oncology cases at the knowledge level of an oncology specialist remains to be tested. Considering performance differences between humans using different summative examination formats and a previous report on the promising accuracy of text-only chatbots in answering patient questions about cancer, this study aims to assess the domain expertise of multimodal chatbots on oncology case questions.^9,10,11^ Compared with text-only chatbots, we benchmarked the medical accuracy of multimodal chatbots on multiple-choice responses and present one of the first benchmarks of multimodal chatbots in generating accurate, free-text responses to questions about cancer vignettes. This study also investigates the utility of prompt engineering (zero-shot chain-of-thought) as an emergent method of designing prompts to optimize the medical accuracy of chatbot responses.^12,13^

## Methods

This cross-sectional study was exempt from approval by the University Health Network Research Ethics Board and exempt from requiring informed consent because no identifiable patient-level data were used and the data were public. We followed the Strengthening the Reporting of Observational Studies in Epidemiology (STROBE) reporting guideline.

We collected all 85 Clinical Challenge oncology cases with quiz questions from JAMA Network Learning accessed on April 2, 2024. Duplicate cases with the same case text, question, and image if available were removed (n = 6), yielding a total set of 79 unique oncology cases included in this study for analysis. The knowledge level of the cases was at the level of oncology specialists. All available images collected as part of each included case were downloaded directly from JAMA Network Learning in the original file format, size, and quality with no manual alteration. We received a Text and Data Mining Policy and License Agreement from the JAMA Network to enter this content into 10 chatbot models (listed below). We carefully reviewed the developer security policies for all the chatbots and adjusted all settings to ensure that none of the JAMA Network content was retained by the models. We evaluated the medical accuracy of 10 chatbots, including ChatGPT-4 Vision (chatbot 1), Claude-3 Sonnet Vision (chatbot 2), Gemini Vision (chatbot 3), ChatGPT-3.5 (chatbot 4), ChatGPT-4 (chatbot 5), Claude-2.1 (chatbot 6), Claude-3 Sonnet (chatbot 7), Gemini (chatbot 8), Llama2 (chatbot 9), and Mistral Large (chatbot 10). This study compared the medical accuracy of the 3 multimodal chatbots (chatbots 1to 3) and 7 unimodal, text-only chatbots (chatbots 4 to 10). Chatbot details are described in eTable 1 in Supplement 1. The clinical cases contained a case description, a question about the case with 4 multiple-choice options, and at least 1 medical image for 72 out of 79 questions. For cases with multiple questions per case, we considered each case and question pair as a standalone query. Out of the 4 multiple-choice options for each case question, there is 1 option determined as the ground-truth answer to the question and used as the reference answer in comparison to chatbot-generated responses to the same question.

Each chatbot response was blinded and randomly ordered before evaluation. All chatbot responses to the accuracy evaluation on oncology cases were conducted on April 6 and 7, 2024. Using a new chatbot session for each case, each chatbot was provided with the case text, question, image if available, multiple-choice options, and prompted to return the correct multiple-choice option in response to the question. Chatbot responses were marked as correct if the multiple-choice option selected was the same as the ground-truth answer or incorrect otherwise. Next, in a new chatbot session for each case, each chatbot was provided with the case text, question, image if available, and prompted to return a free-text response to the question. Chatbot responses were marked as correct or incorrect by 2 independent radiation and medical oncologists based on medical accuracy compared with the ground-truth answer; there were 3 teams of 2 oncologists: (1) D.T. and P.W., (2) J.C. and L.E., and ( 3) M.Y. and A.H. First, oncologist evaluators were instructed to review the case text, question, image if available, and ground-truth multiple-choice answer. Then, oncologist evaluators decided whether the chatbot response was accurate or not compared with the ground-truth multiple-choice answer. Interrater agreement was measured using Cohen κ. Conflicts were resolved by discussion with a third independent evaluator (S.R.) to achieve a consensus decision on the accuracy of the chatbot response. The chatbot prompting procedure is outlined in eFigure 1 in Supplement 1 and zero-shot chain of thought prompt engineering procedure is described in eTable 2 in Supplement 1.^8^

### Statistical Analysis

Statistical analyses were conducted from April 8 to April 19, 2024, using Python version 3.8.9 (Python Software Foundation) with scipy version 1.11.3. χ^2^ test (significance level at *P* < .05) was used to compare the count of correct responses between 2 groups. We used the *t*-test (2-tailed, unpaired, significance level at *P* < .05) to compare the mean word count between correct and incorrect responses for each chatbot.

## Results

Of the 79 questions with multiple-choice options, chatbot 10 was the top-performing chatbot (57 of 79 [72.15%]), followed by multimodal chatbot 2 (56 of 79 [70.89%]) and chatbot 5 (54 of 79 [68.35%]) (Figure, A). We observed performance improvements of chatbot 7 (χ^2^ = 4.15; *P* = .04) when tested on diagnostic questions compared with clinical management questions (Table). Chatbot medical accuracy based on case topics is shown for multiple-choice responses in eTable 3 in Supplement 1 and free-text responses in eTable 4 in Supplement 1.

**Figure.**
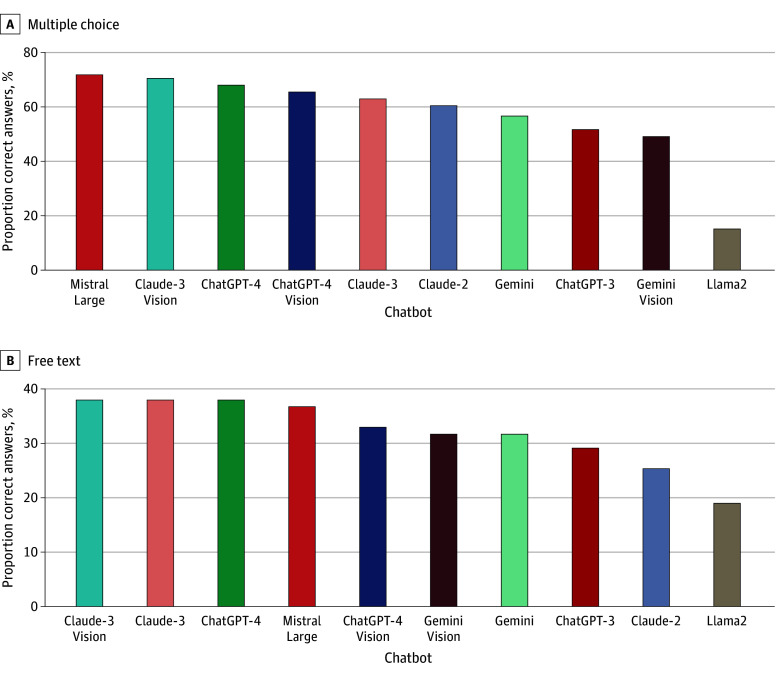
Proportion of Correct Responses to Oncology Case Questions Evaluated Based on Multiple-Choice Response and Free-Text Response Proportion of correct responses out of total number of responses to oncology case questions (N = 79) evaluated based on multiple-choice response (A) and free-text response (B).

**Table.** Medical Accuracy of Chatbots Evaluated on Multiple-Choice and Free-Text Examinations of Clinical Oncology Cases, Stratified by Management and Diagnosis-Related Questions

Chatbot	Multiple choice				Free text			
	No./No. (%)			*P* value^a^	No./No. (%)			*P* value^a^
	Management	Diagnosis	Total		Management	Diagnosis	Total	
Claude-3 Vision	31/42 (73.81)	25/37 (67.57)	56/79 (70.89)	.15	15/42 (35.71)	15/37 (40.54)	30/79 (37.97)	>.99
ChatGPT-4 Vision	23/42 (54.76)	29/37 (78.38)	52/79 (65.82)	.16	14/42 (33.33)	12/37 (32.43)	26/79 (32.91)	.53
Gemini Vision	22/42 (52.38)	17/37 (45.95)	39/79 (49.37)	.17	15/42 (35.71)	10/37 (27.03)	25/79 (31.65)	.09
Mistral Large	32/42 (76.19)	25/37 (67.57)	57/79 (72.15)	.09	14/42 (33.33)	15/37 (40.54)	29/79 (36.71)	.77
ChatGPT-4	24/42 (57.14)	30/37 (81.08)	54/79 (68.35)	.16	17/42 (40.48)	13/37 (35.14)	30/79 (37.97)	.22
ChatGPT-3	24/42 (57.14)	17/37 (45.95)	41/79 (51.9)	.06	13/42 (30.95)	10/37 (27.03)	23/79 (29.11)	.31
Claude-3	29/42 (69.05)	21/37 (56.76)	50/79 (63.29)	.04	18/42 (42.86)	12/37 (32.43)	30/79 (37.97)	.06
Claude-2	24/42 (57.14)	24/37 (64.86)	48/79 (60.76)	>.99	12/42 (28.57)	8/37 (21.62)	20/79 (25.32)	.14
Gemini	26/42 (61.9)	19/37 (51.35)	45/79 (56.96)	.07	14/42 (33.33)	11/37 (29.73)	25/79 (31.65)	.33
Llama2	6/42 (14.29)	6/37 (16.22)	12/79 (15.19)	>.99	9/42 (21.43)	6/37 (16.22)	15/79 (18.99)	.20

^a^ *P* values calculated using χ^2^ test.

Of the 79 questions prompted for free-text responses, the top-performing chatbots were chatbot 5, chatbot 7, and multimodal chatbot 2 (30 of 79 [37.97%]), followed by chatbot 10 (29 of 79 [36.71%]) and chatbot 8 and multimodal chatbot 3 (25 of 79 [31.65%]) (Figure, B). Interrater agreement was moderate to high based on mean Cohen κ (0.55 [95% CI, 0.31-0.79]). No significant difference in accuracy was observed between diagnostic and management questions. In both the multiple-choice and free-text evaluation, the 3 multimodal chatbots varied in accuracy compared with text-only chatbots. For all chatbots except chatbot 9, the accuracy on multiple-choice evaluation was higher than free-text evaluation. Zero-shot chain of thought prompt engineering did not consistently improve chatbot accuracy (eFigure 2 in Supplement 1).

In the multiple-choice evaluation, the majority of noncorrect responses from all 10 chatbots were coded as incorrect (299 of 336 [89.0%]), followed by failure to respond without reason (24 of 336 [7.14%]) and refusal to respond with reason (13 of 336 [3.87%]) (eFigure 3 in Supplement 1). In the free-text evaluation, the majority of noncorrect responses from all 10 chatbots were coded as incorrect (483 of 537 [90.0%]), followed by refusal to respond with reason (44 of 537 [8.19%]) and failure to respond without reason (10 of 537 [1.86%]) (eFigure 3 in Supplement 1). The accuracy of the 3 multimodal chatbots decreased when tested on questions with multiple images compared with questions with single images (eFigure 4 in Supplement 1). Correct chatbot responses generally had equal or longer word counts compared with noncorrect chatbot responses (eFigure 5 in Supplement 1).

## Discussion

Our study prompts a reconsideration of the hypothesis that multimodal AI chatbots, with their broader informational input capabilities, inherently harbor superior medical accuracy.^14^ Notably, chatbot 10, a unimodal chatbot, achieved the highest accuracy in multiple-choice evaluation, while both unimodal (chatbot 5 and 7) and multimodal (chatbot 2) chatbots equally achieved the highest accuracy in free-text evaluation.

The integration of image and text data poses substantial challenges for multimodal AI systems, which must navigate the complexities of processing heterogeneous input data types.^15^ Indeed, we observed that multimodal chatbots performed worse when evaluated on cases with multiple images compared with a single image, suggesting that the quantity of image information may not be associated with medical accuracy. Moreover, the quality and diversity of the training data are critical; suboptimal, or nonrepresentative image components can substantially impair the system’s performance, leading to diagnostic inaccuracies.^16^ AI systems trained on general text and image datasets may find it difficult to generate precise responses to the domain-specific knowledge and reasoning of oncology cases, reflecting the nuanced demands of medical diagnostics in clinical practice.^17^ For instance, benchmarks of AI chatbot performance for specialized tasks in oncology have reported mixed performance, with reported instances of inaccurate generation of chemotherapy protocols with inappropriate duration and dosing,^18^ inconsistent precision oncology recommendations compared with established guidelines,^19^ and hallucinations when used for management recommendations of immune-related adverse events.^20^ Taken together, our results suggest that multimodal AI chatbot applications in oncology may benefit from further evaluation of recurring limitations across specific subtopics to optimize their medical accuracy and reliability.

We found chatbots were less accurate when evaluated on their free-text responses compared with multiple-choice responses. Given that fine-tuning of instructions used to prompt chatbots can improve the multiple-choice accuracy of chatbots, further research to develop instruction prompt tuning for generation of more accurate free-text responses may be a promising direction of clinical chatbot development.^21^ To improve medical accuracy, future development of medical chatbots may also benefit from retrieval-augmented generalization methods to improve accuracy and human alignment to generate realistic, human-like responses.^22^ Moreover, our evaluation of chatbot free-text responses more closely mimics physician-patient interactions in clinical practice, underscoring the importance of realistic clinical assessments of chatbot competencies in medicine.^8^

The inconsistent performance differences due to zero-shot chain-of-thought prompt engineering, demonstrates both the potential and limitations of emergent prompting methodologies to improve AI chatbot reasoning in specialized, domain-specific applications. Although these prompting strategies theoretically offer advantages by steering AI systems toward more reasoned and contextually appropriate responses, more research is needed to ensure reliable improvements in chatbot accuracy across various types of clinical queries and contexts.^23^ A recent study of prompt engineering to improve the reliability of large language models (LLMs) with evidence-based clinical guidelines suggested that evaluation of different combinations of LLMs and prompt engineering methods may optimize LLM-specific performance.^24^ Thus, multimodal AI chatbots may require additional fine-tuning and careful selection of prompt engineering methods in order to take advantage of their multimodal data processing capabilities. To extend our study’s evaluation of zero-shot chain-of-thought prompt engineering to improve chatbot accuracy, future benchmarks of prompt engineering applied to clinical oncology cases can assess promising novel methods of chatbot prompting, such as few-shot heuristic and ensemble prompting.^25^

The application of AI chatbots as a clinical decision support tool requires clinician familiarity and acceptance as critical stakeholders in digital medicine tool design, evaluation, and use.^26^ Clinicians should be able to address patient reservations about clinical AI, including proof of its effectiveness, knowledge of its application, engaging patient participation to identify strengths and weaknesses of clinical chatbot applications, and public accessibility.^27,28^ For multimodal AI chatbot applications, collaboration between clinicians, engineers, and researchers may be needed to identify important features from the rich and heterogeneous multimodal input data used to inform chatbot-generated recommendations.

### Limitations

The primary limitations of this study include the possible inclusion of the study cases in the chatbot training data, the modest case sample size (n = 79), and the high representation of hematological cancer–related case questions (n = 27). In the free-text evaluation, teams of certified oncologists may rate the accuracy of chatbot responses to each case question differently based on their professional judgment and expertise. Development of validated measures of AI chatbot response quality in clinical contexts is a useful next step toward standardized evaluation of chatbot accuracy.^29^ Further evaluation with specific sets of oncology clinician-level cases is needed to compare the performance of unimodal and multimodal chatbots across different facets of oncology and cancer care. Given that chatbots can generate inconsistent responses with the same prompt across independent chatbot sessions, future studies should evaluate the reliability of multimodal chatbot performance across multiple independent replicates.^30^

## Conclusions

In this cross-sectional study of chatbot accuracy, we observed that multimodal chatbots were comparable to unimodal chatbots when evaluated based on accuracy in response to questions about clinical oncology cases and were less accurate when tested on cases with multiple images compared with a single image. Chatbots were generally less accurate when evaluated based on free-text responses compared with multiple-choice responses. Further research is required to improve the reliability of prompt engineering methods to increase accuracy of multimodal chatbots in oncology settings and evaluate the utility of AI chatbots as useful decision support tools in clinical oncology workflows.

## Notes

Supplement 1. **eFigure 1.** Prompting Procedure of Text-Only and Multi-Modal Chatbots **eFigure 2.** Performance of Prompt-Engineering and Zero-Shot Evaluation of Chatbot Performance on Multiple-choice and Free-text Evaluations **eFigure 3**. Categorization of Reasons for Incorrect Chatbot Responses **eFigure 4.** Association of the Number of Case Images and Performance of Chatbots on Questions About Cancer Clinical Cases **eFigure 5.** Association of Word Count and Performance of Chatbots on Questions About Cancer Clinical Cases **eTable 1**. Description of Chatbot Models Evaluated in This Study **eTable 2.** Zero-Shot Chain-of-Thought (CoT) Prompt-Engineering Procedure **eTable 3.** Multiple-Choice Performance of Chatbots on Questions About Cancer Clinical Cases, Split by Medical Topic **eTable 4.** Free-Text Performance of Chatbots on Questions About Cancer Clinical Cases, Split by Medical Topic **eMethods.** Instructions to Physician Raters

Supplement 2.
Data Sharing Statement
