# Supplementary material for: Performance of Multimodal Artificial Intelligence Chatbots Evaluated on Clinical Oncology Cases
Source: JAMA Netw Open. 2024 Oct 23;7(10):e2437711. doi: 10.1001/jamanetworkopen.2024.37711 (PMC11581577; doi:10.1001/jamanetworkopen.2024.37711)
Supplement: Supplement 1. — eFigure 1. Prompting Procedure of Text-Only and Multi-Modal Chatbots eFigure 2. Performance of Prompt-Engineering and Zero-Shot Evaluation of Chatbot Performance on Multiple-choice and Free-text Evaluations eFigure 3. Categorization of Reasons for Incorrect Chatbot Responses eFigure 4. Association of the Number of Case Images and Performance of Chatbots on Questions About Cancer Clinical Cases eFigure 5. Association of Word Count and Performance of Chatbots on Questions About Cancer Clinical Cases eTable 1. Description of Chatbot Models Evaluated in This Study eTable 2. Zero-Shot Chain-of-Thought (CoT) Prompt-Engineering Procedure eTable 3. Multiple-Choice Performance of Chatbots on Questions About Cancer Clinical Cases, Split by Medical Topic eTable 4. Free-Text Performance of Chatbots on Questions About Cancer Clinical Cases, Split by Medical Topic eMethods. Instructions to Physician Raters [file jamanetwopen-e2437711-s001.pdf]

Supplemental Online Content

Chen D, Huang RS, Jomy J, et al. Performance of multimodal artificial intelligence chatbots evaluated on clinical oncology cases. *JAMA Netw Open.* 2024;7(10):e2437711. doi:10.1001/jamanetworkopen.2024.37711

Supplement 1. eFigure 1. Prompting Procedure of Text-Only and Multi-Modal Chatbots

eFigure 2. Performance of Prompt-Engineering and Zero-Shot Evaluation of Chatbot Performance on Multiple-choice and Free-text Evaluations

eFigure 3 . Categorization of Reasons for Incorrect Chatbot Responses

eFigure 4. Association of the Number of Case Images and Performance of Chatbots on Questions About Cancer Clinical Cases

eFigure 5. Association of Word Count and Performance of Chatbots on Questions About Cancer Clinical Cases

eTable 1 . Description of Chatbot Models Evaluated in This Study

eTable 2. Zero-Shot Chain-of-Thought (CoT) Prompt-Engineering Procedure

eTable 3. Multiple-Choice Performance of Chatbots on Questions About Cancer Clinical Cases, Split by Medical Topic

eTable 4. Free-Text Performance of Chatbots on Questions About Cancer Clinical Cases, Split by Medical Topic

eMethods. Instructions to Physician Raters

This supplemental material has been provided by the authors to give readers additional information about their work.

eFigure 1. Prompting Procedure of Text-only and Multi-modal Chatbots.

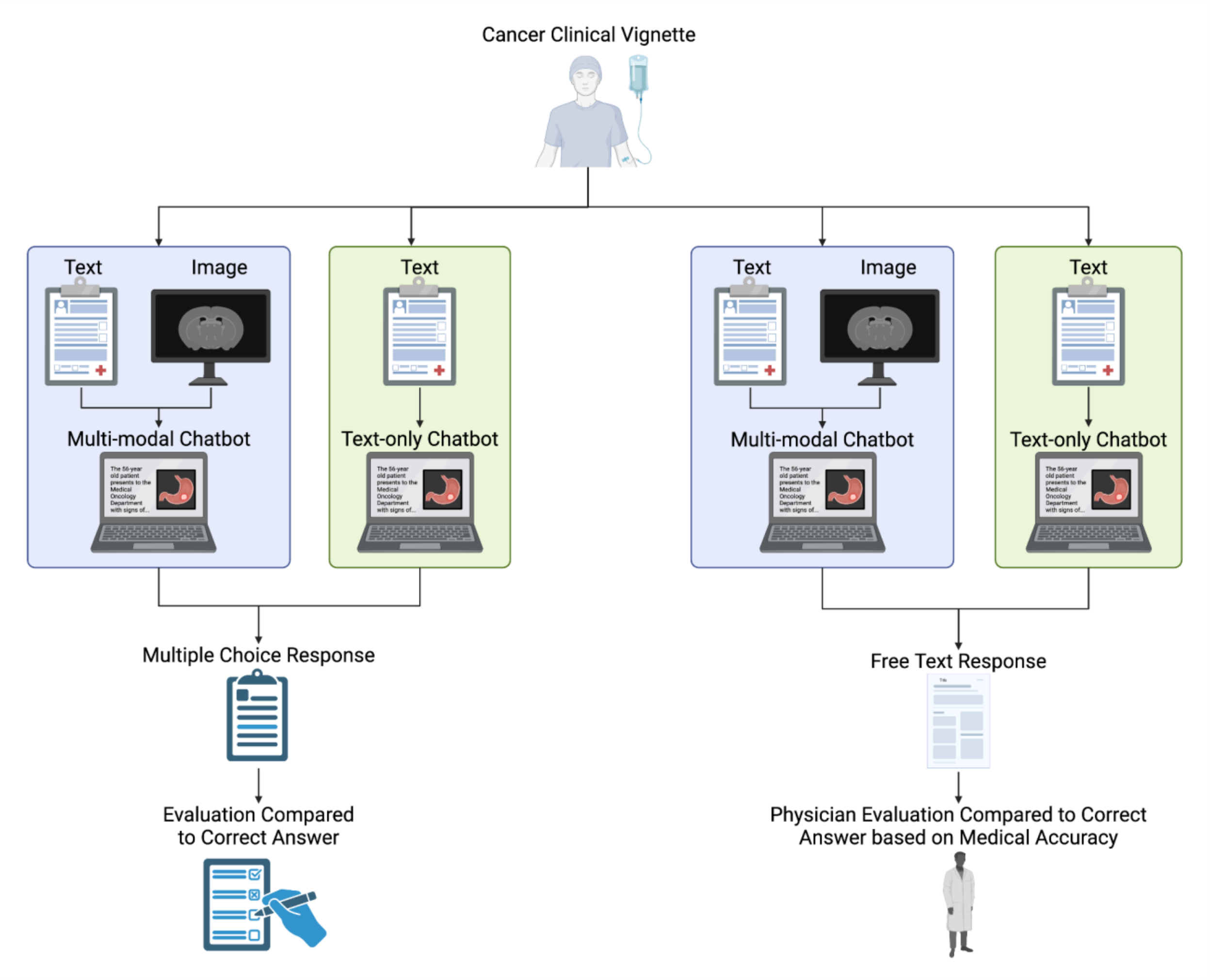

© 2024 Chen D et al. *JAMA Network Open.*

eFigure 2. Performance of Prompt-engineering and Zero-shot Evaluation of Chatbot Performance on Multiple-choice and Free-text Evaluations.

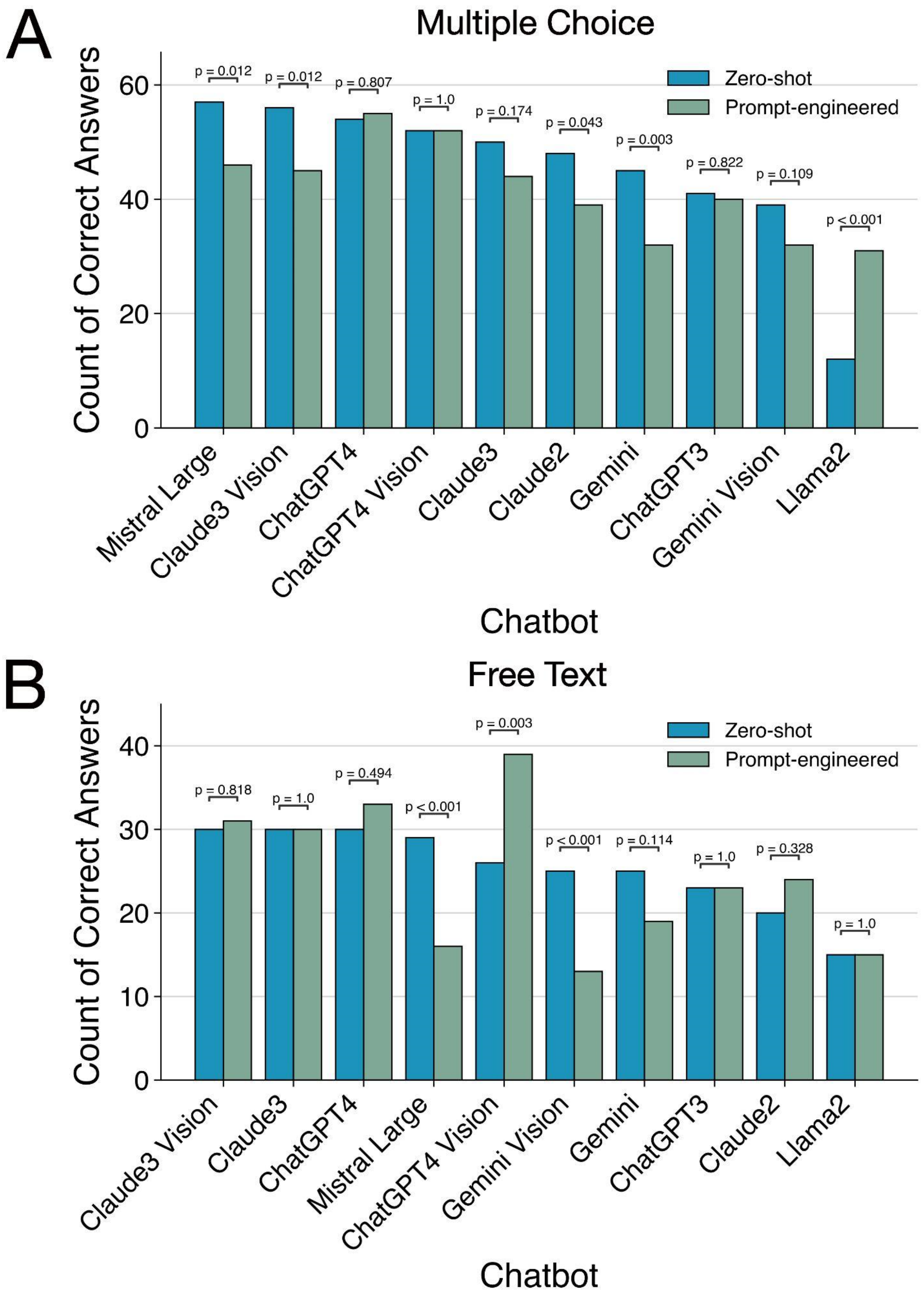

© 2024 Chen D et al. *JAMA Network Open.*

eFigure 3. Categorization of Reasons for Incorrect Chatbot Responses. Incorrect responses were coded as Refuse to Answer if the chatbot declined to respond with a reason. Incorrect responses were coded as Nonsense if the chatbot declined to respond with no reason or provided a technical error that prohibited the generation of a response.

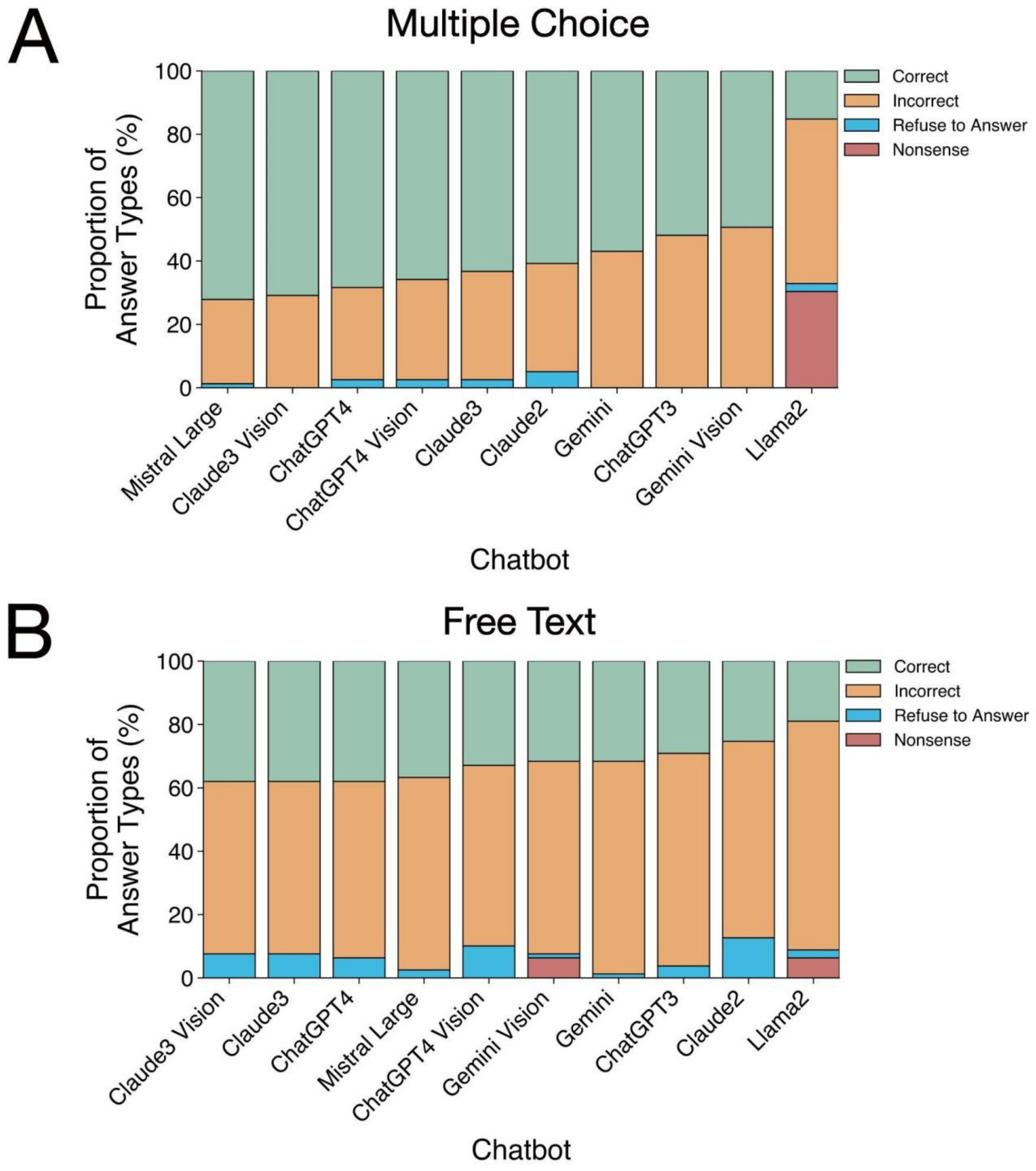

© 2024 Chen D et al. *JAMA Network Open.*

eFigure 4. Association of the Number of Case Images and Performance of Chatbots on Questions about Cancer Clinical Cases.

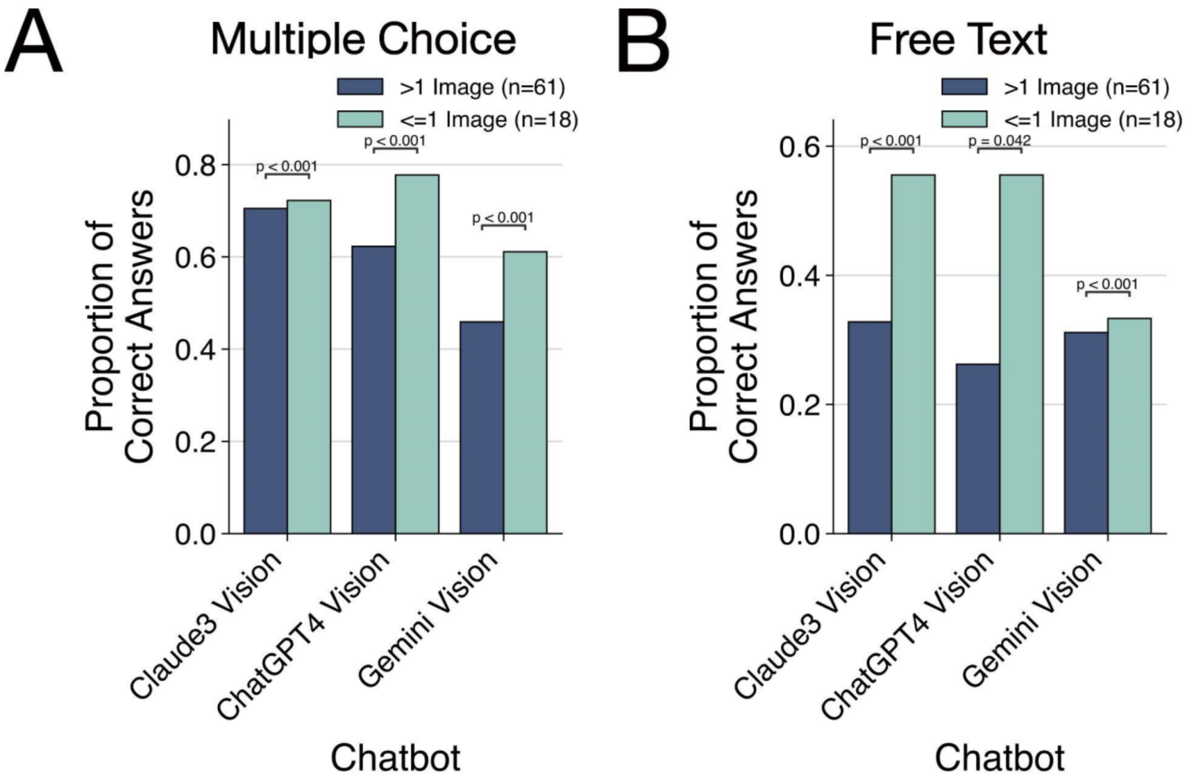

© 2024 Chen D et al. *JAMA Network Open.*

eFigure 5. Association of Word Count and Performance of Chatbots on Questions about Cancer Clinical Cases (* P<0.05).

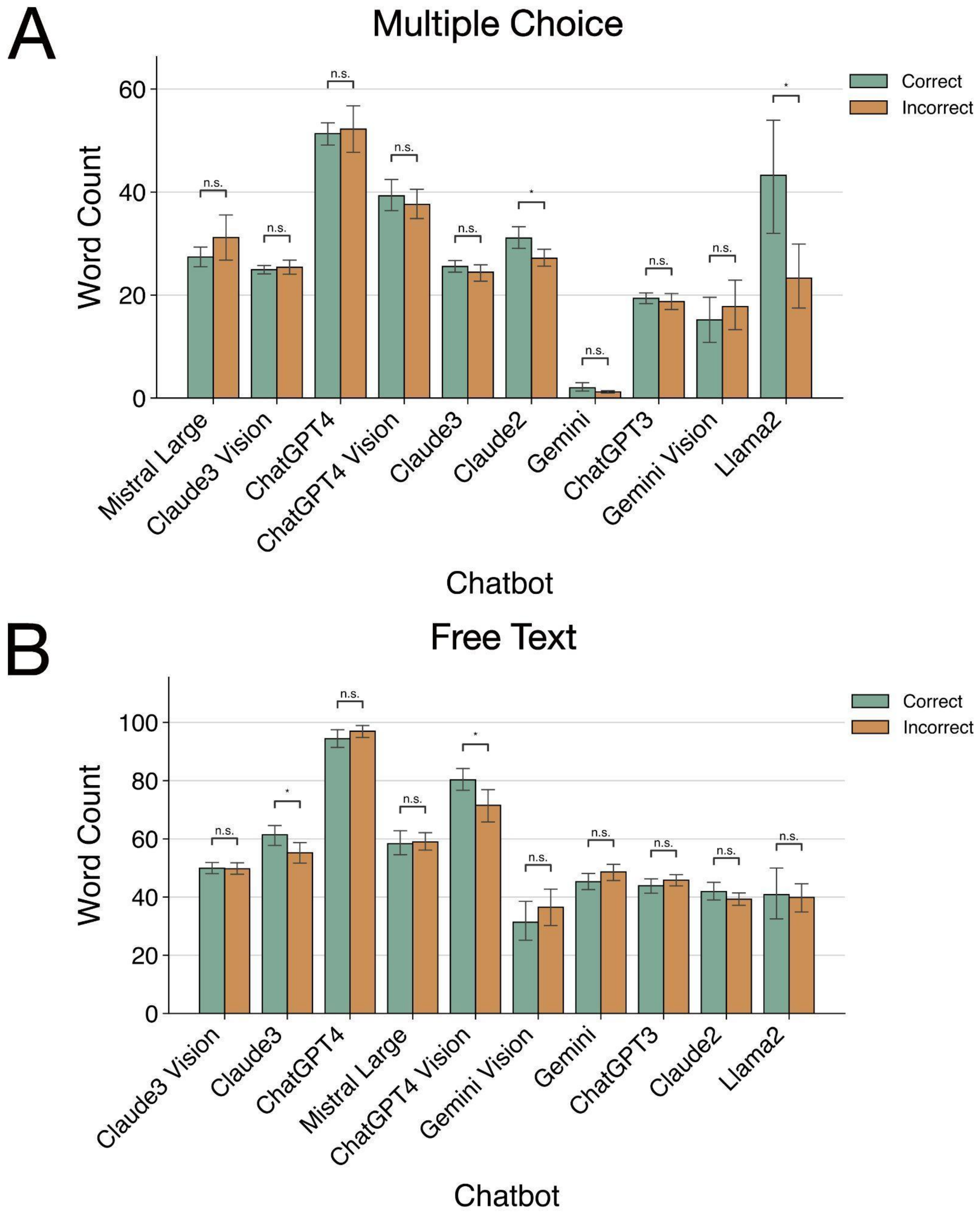

© 2024 Chen D et al. *JAMA Network Open.*

eTable 1. Description of Chatbot Models Evaluated in this Study.

**Chatbot**	**Developer**	**Release Date**	**API Access**	**API Model ID**	**Input Type**
Mistral Large	Mistral AI	February 26, 2024	Amazon Bedrock	mistral.mistral-large-2402-v1:0	Text
Claude3 Sonnet	Anthropic	March 4, 2024	Amazon Bedrock	anthropic.claude-3-sonnet-20240229-v1:0	Text
Claude3 Sonnet Vision	Anthropic	March 4, 2024	Amazon Bedrock	anthropic.claude-3-sonnet-20240229-v1:0	Text and Image
ChatGPT4	OpenAI	March 14, 2023	OpenAI	gpt-4-0125-preview	Text
ChatGPT4 Vision	OpenAI	September 25, 2023	OpenAI	gpt-4-0125-preview	Text and Image
Claude 2.1	Anthropic	November 21, 2023	Amazon Bedrock	anthropic.claude-v2:1	Text
Gemini Pro	Google	December 6, 2023	Google	gemini-pro	Text
Gemini Pro Vision	Google	December 6, 2023	Google	gemini-pro-vision	Text and Image
ChatGPT3.5	OpenAI	November 30, 2022	OpenAI	gpt-3.5-turbo	Text
Llama2 70B	Meta and Microsoft	July 18, 2023	Amazon Bedrock	meta.llama2-70b-chat-v1	Text

eTable 2. Zero-shot Chain-of-thought (CoT) Prompt-engineering Procedure.

Prompt ID	Prompt
CoT 1	You are an oncology medical professional. Your goal is to generate high quality, empathetic, and readable responses to patients' questions about cancer. The intended audience is patients or their associates who are experiencing cancer.
CoT 2	Identify which sections of the clinical vignette and image (if available) are important to answer the patient question about cancer
CoT 3	Medical accuracy is defined as being supported by research conducted in compliance with scientific methods and published in peer-reviewed journals, where appropriate, and recognized as accurate and objective by professional organizations and agencies with expertise in the relevant field. Reason how you can integrate the important sections of the clinical vignette and image (if available) to improve the medical accuracy of your response to the question about the clinical vignette. Think step by step.
Multiple Choice Question	As an oncology medical professional, select one of the multiple choice options (A, B, C, or D) in response to the clinical vignette. Provide the selected multiple choice option as the first sentence of your response.
Free Text Question	As an oncology medical professional, provide medical recommendations related to diagnosis, management, and treatment in response to the patient question about cancer.

© 2024 Chen D et al. *JAMA Network Open.*

eTable 3. Multiple-choice Performance of Chatbots on Questions about Cancer Clinical Vignettes, split by medical topic.

	Claude3 Vision	ChatGPT 4 Vision	Gemini Vision	Mistral Large	ChatGPT 4	ChatGPT 3	Claude3	Claude2	Gemini	Llama2
**Hematologic Cancer**	15/27 (55.56%)	17/27 (62.96%)	10/27 (37.04%)	16/27 (59.26%)	17/27 (62.96%)	11/27 (40.74%)	12/27 (44.44%)	16/27 (59.26%)	15/27 (55.56%)	2/27 (7.41%)
**Surgical Oncology**	5/7 (71.43%)	4/7 (57.14%)	5/7 (71.43%)	6/7 (85.71%)	4/7 (57.14%)	6/7 (85.71%)	5/7 (71.43%)	6/7 (85.71%)	4/7 (57.14%)	1/7 (14.29%)
**Breast Cancer**	5/7 (71.43%)	3/7 (42.86%)	5/7 (71.43%)	6/7 (85.71%)	3/7 (42.86%)	4/7 (57.14%)	5/7 (71.43%)	5/7 (71.43%)	3/7 (42.86%)	1/7 (14.29%)
**Skin Cancer**	6/6 (100.0%)	6/6 (100.0%)	2/6 (33.33%)	5/6 (83.33%)	6/6 (100.0%)	4/6 (66.67%)	6/6 (100.0%)	4/6 (66.67%)	3/6 (50.0%)	1/6 (16.67%)
**Gastrointestinal Cancer**	4/5 (80.0%)	4/5 (80.0%)	3/5 (60.0%)	5/5 (100.0%)	5/5 (100.0%)	4/5 (80.0%)	4/5 (80.0%)	2/5 (40.0%)	4/5 (80.0%)	2/5 (40.0%)
**Urologic Cancer**	4/5 (80.0%)	3/5 (60.0%)	4/5 (80.0%)	4/5 (80.0%)	3/5 (60.0%)	0/5 (0.0%)	3/5 (60.0%)	4/5 (80.0%)	3/5 (60.0%)	1/5 (20.0%)
**Radiation Oncology**	3/4 (75.0%)	2/4 (50.0%)	2/4 (50.0%)	3/4 (75.0%)	2/4 (50.0%)	3/4 (75.0%)	2/4 (50.0%)	2/4 (50.0%)	4/4 (100.0%)	0/4 (0.0%)
**Head and Neck Cancer**	2/3 (66.67%)	1/3 (33.33%)	1/3 (33.33%)	2/3 (66.67%)	2/3 (66.67%)	2/3 (66.67%)	2/3 (66.67%)	2/3 (66.67%)	2/3 (66.67%)	2/3 (66.67%)
**Cancer Genetics**	2/3 (66.67%)	3/3 (100.0%)	1/3 (33.33%)	2/3 (66.67%)	3/3 (100.0%)	2/3 (66.67%)	2/3 (66.67%)	2/3 (66.67%)	2/3 (66.67%)	0/3 (0.0%)
**Skin Cancer, Targeted and Immune Therapy**	1/2 (50.0%)	1/2 (50.0%)	1/2 (50.0%)	0/2 (0.0%)	1/2 (50.0%)	1/2 (50.0%)	1/2 (50.0%)	0/2 (0.0%)	1/2 (50.0%)	0/2 (0.0%)
**Gastrointestinal Cancer, Targeted and Immune Therapy**	2/2 (100.0%)	2/2 (100.0%)	2/2 (100.0%)	2/2 (100.0%)	2/2 (100.0%)	1/2 (50.0%)	1/2 (50.0%)	2/2 (100.0%)	2/2 (100.0%)	0/2 (0.0%)
**Gynecologic Cancer**	2/2 (100.0%)	1/2 (50.0%)	0/2 (0.0%)	0/2 (0.0%)	1/2 (50.0%)	0/2 (0.0%)	2/2 (100.0%)	0/2 (0.0%)	0/2 (0.0%)	0/2 (0.0%)
**Cancer Screening, Prevention, Control**	1/1 (100.0%)	1/1 (100.0%)	1/1 (100.0%)	1/1 (100.0%)	1/1 (100.0%)	1/1 (100.0%)	1/1 (100.0%)	1/1 (100.0%)	1/1 (100.0%)	1/1 (100.0%)

© 2024 Chen D et al. *JAMA Network Open.*

**Hematologic Cancer, Skin Cancer, Targeted and Immune Therapy**	0/1 (0.0%)	0/1 (0.0%)	0/1 (0.0%)	1/1 (100.0%)	0/1 (0.0%)	0/1 (0.0%)	0/1 (0.0%)	0/1 (0.0%)	0/1 (0.0%)	0/1 (0.0%)
**Breast Cancer, Gastrointestinal Cancer**	1/1 (100.0%)	1/1 (100.0%)	1/1 (100.0%)	1/1 (100.0%)	1/1 (100.0%)	1/1 (100.0%)	1/1 (100.0%)	1/1 (100.0%)	0/1 (0.0%)	0/1 (0.0%)
**Targeted and Immune Therapy**	1/1 (100.0%)	1/1 (100.0%)	0/1 (0.0%)	1/1 (100.0%)	1/1 (100.0%)	0/1 (0.0%)	1/1 (100.0%)	1/1 (100.0%)	0/1 (0.0%)	0/1 (0.0%)
**Targeted and Immune Therapy, Lung Cancer**	1/1 (100.0%)	1/1 (100.0%)	0/1 (0.0%)	1/1 (100.0%)	1/1 (100.0%)	1/1 (100.0%)	1/1 (100.0%)	0/1 (0.0%)	0/1 (0.0%)	1/1 (100.0%)
**Lung Cancer**	1/1 (100.0%)	1/1 (100.0%)	1/1 (100.0%)	1/1 (100.0%)	1/1 (100.0%)	0/1 (0.0%)	1/1 (100.0%)	0/1 (0.0%)	1/1 (100.0%)	0/1 (0.0%)
**Total**	56/79 (70.89%)	52/79 (65.82%)	39/79 (49.37%)	57/79 (72.15%)	54/79 (68.35%)	41/79 (51.9%)	50/79 (63.29%)	48/79 (60.76%)	45/79 (56.96%)	12/79 (15.19%)

© 2024 Chen D et al. *JAMA Network Open.*

eTable 4. Free-text Performance of Chatbots on Questions about Cancer Clinical Vignettes, split by medical topic.

	Claude3 Vision	Claude3	ChatGPT 4	Mistral Large	ChatGPT 4 Vision	Gemini Vision	Gemini	ChatGPT 3	Claude2	Llama2
Hematologic Cancer	9/27 (33.33%)	9/27 (33.33%)	8/27 (29.63%)	12/27 (44.44%)	9/27 (33.33%)	8/27 (29.63%)	6/27 (22.22%)	7/27 (25.93%)	6/27 (22.22%)	6/27 (22.22%)
Surgical Oncology	3/7 (42.86%)	5/7 (71.43%)	2/7 (28.57%)	2/7 (28.57%)	3/7 (42.86%)	3/7 (42.86%)	4/7 (57.14%)	2/7 (28.57%)	2/7 (28.57%)	2/7 (28.57%)
Breast Cancer	3/7 (42.86%)	3/7 (42.86%)	2/7 (28.57%)	1/7 (14.29%)	2/7 (28.57%)	1/7 (14.29%)	1/7 (14.29%)	3/7 (42.86%)	2/7 (28.57%)	1/7 (14.29%)
Skin Cancer	2/6 (33.33%)	3/6 (50.0%)	3/6 (50.0%)	2/6 (33.33%)	2/6 (33.33%)	2/6 (33.33%)	3/6 (50.0%)	2/6 (33.33%)	2/6 (33.33%)	1/6 (16.67%)
Gastrointestinal Cancer	4/5 (80.0%)	2/5 (40.0%)	4/5 (80.0%)	3/5 (60.0%)	4/5 (80.0%)	3/5 (60.0%)	3/5 (60.0%)	4/5 (80.0%)	2/5 (40.0%)	1/5 (20.0%)
Urologic Cancer	0/5 (0.0%)	0/5 (0.0%)	1/5 (20.0%)	1/5 (20.0%)	1/5 (20.0%)	1/5 (20.0%)	0/5 (0.0%)	0/5 (0.0%)	0/5 (0.0%)	1/5 (20.0%)
Radiation Oncology	1/4 (25.0%)	0/4 (0.0%)	2/4 (50.0%)	2/4 (50.0%)	1/4 (25.0%)	1/4 (25.0%)	2/4 (50.0%)	0/4 (0.0%)	1/4 (25.0%)	0/4 (0.0%)
Head and Neck Cancer	1/3 (33.33%)	1/3 (33.33%)	0/3 (0.0%)	0/3 (0.0%)	0/3 (0.0%)	1/3 (33.33%)	0/3 (0.0%)	1/3 (33.33%)	0/3 (0.0%)	1/3 (33.33%)
Cancer Genetics	1/3 (33.33%)	2/3 (66.67%)	2/3 (66.67%)	1/3 (33.33%)	2/3 (66.67%)	1/3 (33.33%)	2/3 (66.67%)	2/3 (66.67%)	2/3 (66.67%)	1/3 (33.33%)
Skin Cancer, Targeted and Immune Therapy	1/2 (50.0%)	1/2 (50.0%)	0/2 (0.0%)	1/2 (50.0%)	0/2 (0.0%)	0/2 (0.0%)	0/2 (0.0%)	0/2 (0.0%)	0/2 (0.0%)	0/2 (0.0%)
Gastrointestinal Cancer, Targeted and Immune Therapy	2/2 (100.0%)	1/2 (50.0%)	1/2 (50.0%)	1/2 (50.0%)	1/2 (50.0%)	2/2 (100.0%)	1/2 (50.0%)	0/2 (0.0%)	0/2 (0.0%)	0/2 (0.0%)
Gynecologic Cancer	1/2 (50.0%)	1/2 (50.0%)	1/2 (50.0%)	0/2 (0.0%)	0/2 (0.0%)	0/2 (0.0%)	0/2 (0.0%)	0/2 (0.0%)	1/2 (50.0%)	0/2 (0.0%)
Cancer Screening, Prevention, Control	1/1 (100.0%)	1/1 (100.0%)	1/1 (100.0%)	1/1 (100.0%)	1/1 (100.0%)	0/1 (0.0%)	1/1 (100.0%)	1/1 (100.0%)	1/1 (100.0%)	1/1 (100.0%)

© 2024 Chen D et al. *JAMA Network Open.*

**Hematologic Cancer, Skin Cancer, Targeted and Immune Therapy**	0/1 (0.0%)	0/1 (0.0%)	0/1 (0.0%)	0/1 (0.0%)	0/1 (0.0%)	0/1 (0.0%)	1/1 (100.0%)	0/1 (0.0%)	0/1 (0.0%)	0/1 (0.0%)
**Breast Cancer, Gastrointestinal Cancer**	0/1 (0.0%)	0/1 (0.0%)	0/1 (0.0%)	0/1 (0.0%)	0/1 (0.0%)	0/1 (0.0%)	1/1 (100.0%)	1/1 (100.0%)	0/1 (0.0%)	0/1 (0.0%)
**Targeted and Immune Therapy**	1/1 (100.0%)	1/1 (100.0%)	1/1 (100.0%)	1/1 (100.0%)	0/1 (0.0%)	1/1 (100.0%)	0/1 (0.0%)	0/1 (0.0%)	0/1 (0.0%)	0/1 (0.0%)
**Targeted and Immune Therapy, Lung Cancer**	0/1 (0.0%)	0/1 (0.0%)	1/1 (100.0%)	1/1 (100.0%)	0/1 (0.0%)	0/1 (0.0%)	0/1 (0.0%)	0/1 (0.0%)	0/1 (0.0%)	0/1 (0.0%)
**Lung Cancer**	0/1 (0.0%)	0/1 (0.0%)	1/1 (100.0%)	0/1 (0.0%)	0/1 (0.0%)	1/1 (100.0%)	0/1 (0.0%)	0/1 (0.0%)	1/1 (100.0%)	0/1 (0.0%)
**Total**	30/79 (37.97%)	30/79 (37.97%)	30/79 (37.97%)	29/79 (36.71%)	26/79 (32.91%)	25/79 (31.65%)	25/79 (31.65%)	23/79 (29.11%)	20/79 (25.32%)	15/79 (18.99%)

© 2024 Chen D et al. *JAMA Network Open.*

eMethods. Instructions to Physician Raters

Introduction

Thank you for agreeing to participate in this project as a co-author collaborator. This project is focused on evaluating chatbot competency in responding to questions about cancer clinical vignettes. Dr. Srinivas Raman and David Chen are incredibly grateful for your time and expertise in moving forward this part of the study.

Study Novelty

This study is the first to evaluate:

1) The medical accuracy of **multi-modal** and text-only chatbots in response to **cancer clinical vignettes**
2) The utility of **prompt engineering** as a novel communication methodology to improve the medical accuracy of chatbots
3) The medical accuracy of **free-text** chatbot responses in comparison to classical multiple choice chatbot responses

Background

Classic benchmarks of chatbot medical accuracy have prompted chatbots to respond to multiple-choice exam style questions. However, given that a clinical vignette more accurately mimics real-world clinical scenarios than knowledge/recall-based multiple choice questions, it would be more realistic to test the medical accuracy of free-text chatbot responses than selection from a provided list of multiple choice questions. Real-world patient presentations do not automatically provide you with differential options to select from; instead, competent clinicians would need to arrive at the most correct answer through independent evidence-based reasoning and logic.

Objective

The primary aim of this phase of the study is to rate the performance of free-text chatbot responses to questions about cancer clinical vignettes.

Dataset Description

The total dataset includes a set of 79 clinical vignettes with 1) text about the cancer vignette, 2) question related to the vignette, and 3) chatbot responses to the question. Chatbots were prompted to respond to each question using two approaches: 1) zero-shot direct response to the question and 2) prompt-engineered response to the question. For more information about prompt engineering, please refer to this resource.

For your assigned set of ratings, you will receive clinical vignettes with associated questions and chatbot responses.

© 2024 Chen D et al. *JAMA Network Open.*

Rating Steps

Step	Task
1	Read the ground-truth, correct answer (column D) to the vignette question (column C). It may be helpful to read the vignette text (column B) if necessary.
2	For each chatbot, note that there is one column for the chatbot's response and one neighboring column for the clinician's rating of the medical accuracy of the response.<br><br>Using the drop-down menu, select one of four options to assess the chatbot's response:<br>1. Correct: The free-text response includes each element of the ground-truth answer<br>2. Incorrect: The free-text response does not include each element of the ground-truth answer<br>3. Refuse to Answer: The free-text response states that it cannot answer the question and provides a specified reason.<br>4. Nonsense: The free-text response is non-sensical, not accurate, and did not provide a reason why it could not answer the question

Note 1: In some instances, ground-truth answers will have **2+ recommendations**. Correct chatbot responses **must contain each element of the ground-truth, correct answer**. If the chatbot response only contains 1 out of the 2 ground-truth recommendations, it should be marked as **incorrect**.

Note 2: Although assessment of medical accuracy is judgment-based, a chatbot response marked as correct **does not need to word-for-word replicate the ground truth answer**. If you feel that the chatbot response mimics the intention, reasoning, and expected outcome associated with the ground truth answer and is generally clinically acceptable/reasonable, you can mark the response as correct based on your clinical judgment.

© 2024 Chen D et al. *JAMA Network Open.*

Examples of each of the four ratings and chatbot responses

Correct Answer: C. Biopsy the EOMs

Rating	Chatbot Response Example
Correct	Obtain a biopsy of the extraocular muscles to confirm the diagnosis and rule out other potential causes, such as thyroid eye disease or inflammatory myopathies.
Incorrect	Start intravenous octreotide to help control symptoms of flushing and reduce vascular permeability leading to chemosis. This can rapidly improve vision and comfort.
Refuse to Answer	This patient is clearly undergoing extensive medical evaluation and I do not have enough information to make specific recommendations about her diagnosis, management or treatment.
Nonsense (also described as Failed/Blocked/Error)	Failed: The \`response.parts\` quick accessor only works for a single candidate, but none were returned. Check the \`response.prompt_feedback\` to see if the prompt was blocked.

© 2024 Chen D et al. *JAMA Network Open.*
