# Supplementary material for: Performance of Multimodal Artificial Intelligence Chatbots Evaluated on Clinical Oncology Cases
Source: JAMA Netw Open. 2024 Oct 23;7(10):e2437711. doi: 10.1001/jamanetworkopen.2024.37711 (PMC11581577; doi:10.1001/jamanetworkopen.2024.37711)
Supplement: Supplement 2. — Data Sharing Statement [file jamanetwopen-e2437711-s002.pdf]

Data Sharing Statement

Chen. Performance of Multimodal Artificial Intelligence Chatbots Evaluated on Clinical Oncology Cases. *JAMA Netw Open*. Published October 04, 2024.
doi:10.1001/jamanetworkopen.2024.37711

Data
Data available: Yes
Data types: Data (not involving human participants)
How to access data: Data will be shared via email requests to the corresponding author: srinivas.raman@uhn.ca
When available: With publication

Supporting Documents
Document types: None

Additional Information
Who can access the data: anyone requesting the data
Types of analyses: any purpose
Mechanisms of data availability: with investigator support
Any additional restrictions: None
